# P-1715. Fluconazole Minimum Inhibitory Concentration in Cryptococcus Infections and its Association with Survival

**DOI:** 10.1093/ofid/ofaf695.1887

**Published:** 2026-01-11

**Authors:** Deepali Boothankad Sharath, Kyle D Brizendine, Anisha Misra

**Affiliations:** Cleveland Clinic Foundation, Cleveland, OH; Cleveland Clinic Foundation, Cleveland, OH; Cleveland Clinic Foundation, Cleveland, OH

## Abstract

**Background:**

There are concerns about fluconazole resistance in cryptococcosis and the impact on patient outcomes. At present, there is insufficient data to suggest fluconazole minimum inhibitory concentration (MIC) independently affects outcomes.

**Figure:**
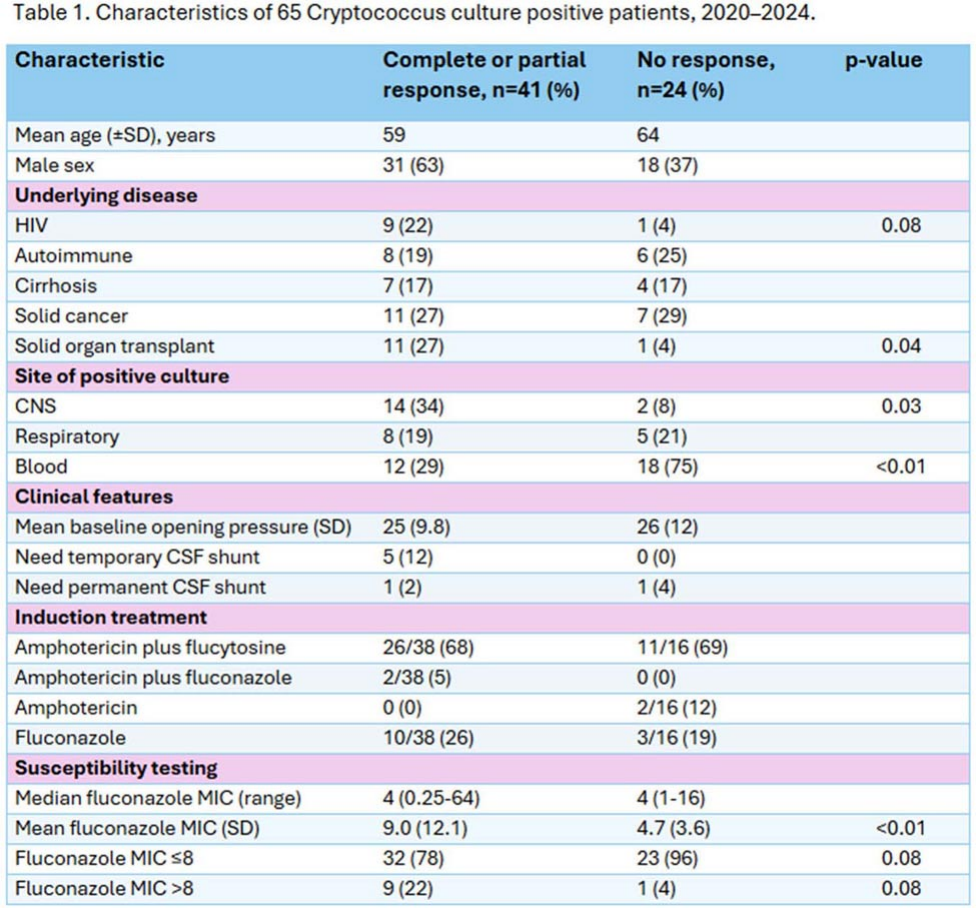

**Figure:**
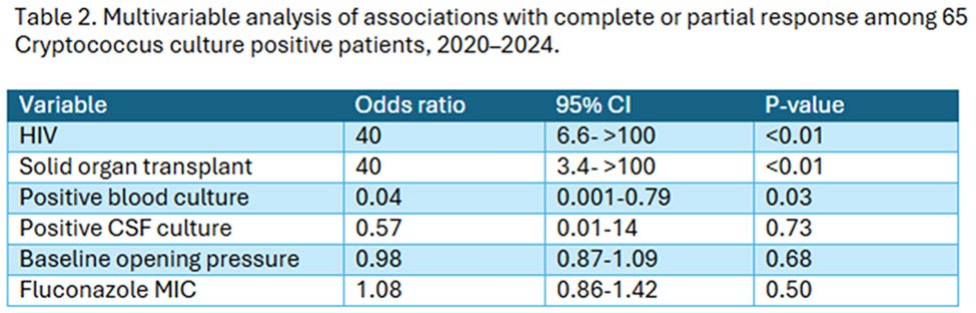

**Methods:**

This was a retrospective, single center observational cohort study. We analyzed clinical and microbiological data from patients with culture confirmed *Cryptococcus* infections over a 5– year period (2020-2024) to determine the association between MIC and outcome, defined according to EORTC/MSG consensus criteria for responses to therapy at 12 weeks. MIC was determined by broth microdilution. The Kaplan-Meier method and multivariable logistic regression were utilized.

**Figure:**
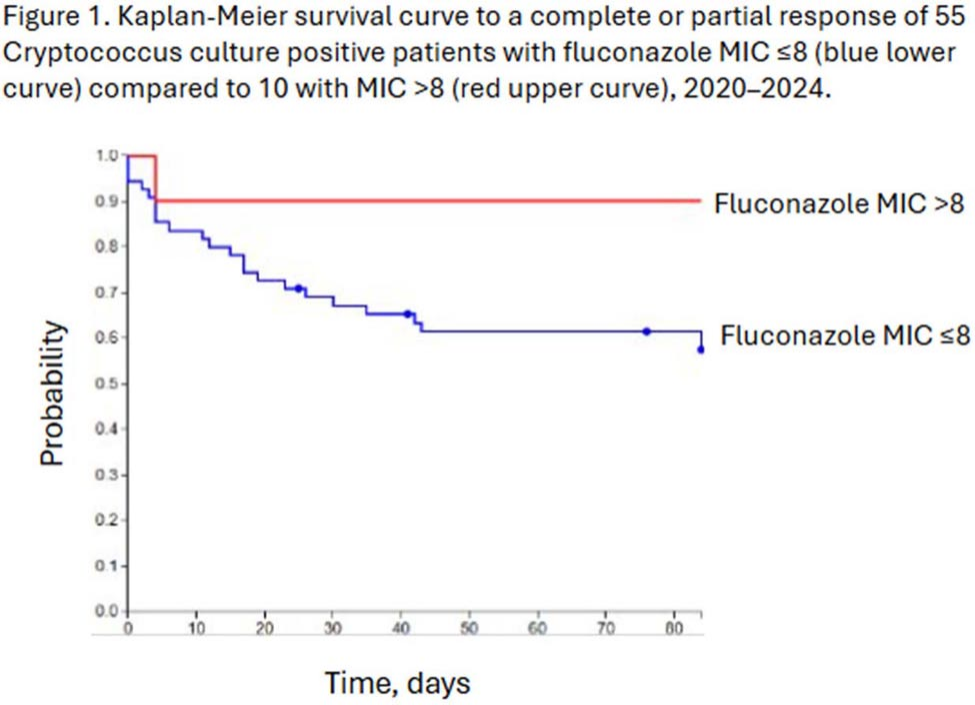

**Results:**

We identified 82 patients with culture positive *Cryptococcus* infections, all were neoformans; 65 had MIC data and were assessable for response: 41 (63%; 95% CI 51–74%) had complete or partial response and 24 (37%; 95% CI 26-49%) no response. Table 1 shows the characteristics. There was no difference in proportion receiving induction with amphotericin B plus flucytosine in both groups (68 vs. 69%), following which 61/65 received fluconazole. All-cause mortality was 22/65 (34%; 95% CI 24–46%). Median fluconazole MIC increased over time from 2 to 8 in the periods 2020-2022 to 2023-2024, respectively. Using multivariable logistic regression analyses, as reported in Table 2, increased odds of complete or partial response were observed with HIV and solid organ transplant (SOT) compared to other underlying diseases, and decreased odds of response were seen in patients with positive blood cultures. Importantly, fluconazole MIC was not associated with response at 12 weeks. Figure 1 shows the Kaplan-Meier survival curve with no difference in survival between patients with MIC ≤ 8 compared to patients with MIC > 8.

**Conclusion:**

Higher MIC was not associated with poor outcomes; however, we did note an increase in median MIC over time. Ongoing MIC surveillance and assessment of MIC’s impact on outcomes in future studies will be important. The present study showed cryptococcemia to be a strong predictor of no response to therapy while HIV and SOT status was associated with response to therapy. Therefore, bloodstream infection and underlying disease may be more prognostic than MIC.

**Disclosures:**

Kyle D. Brizendine, MD, Pfizer: Advisor/Consultant

